# Intracytoplasmic Sperm Injection Using 20-Year-Old Cryopreserved Sperm Results in Normal, Viable, and Reproductive Offspring in *Xenopus laevis*: A Major Pioneering Achievement for Amphibian Conservation

**DOI:** 10.3390/ani15131941

**Published:** 2025-07-01

**Authors:** Louise Péricard, Sébastien Le Mével, Olivier Marquis, Yann Locatelli, Laurent Coen

**Affiliations:** 1 Physiologie Moléculaire et Adaptation, UMR 7221 Centre National de la Recherche Scientifique, Muséum National d’Histoire Naturelle, Cedex 05, 75231 Paris, Francelaurent.coen@mnhn.fr (L.C.); 2 Parc Zoologique de Paris, Muséum National d’Histoire Naturelle, 75012 Paris, France; olivier.marquis@mnhn.fr; 3 Réserve Zoologique de la Haute Touche, Muséum National d’Histoire Naturelle, 36290 Obterre, France

**Keywords:** amphibian decline, *Xenopus laevis*, sperm cryopreservation, intracytoplasmic sperm injection, in vitro fertilization

## Abstract

The significant decline in amphibians worldwide is demanding the development of reliable techniques to save species and their genetic diversity. Considerable efforts are currently in progress to develop assisted reproductive technologies (ARTs), focusing mainly on sperm cryopreservation and in vitro fertilization (IVF). In *Xenopus*, a simple and efficient transgenesis method based on the intracytoplasmic injection (ICSI) of cryoconserved sperm was developed several decades ago, allowing for quick generation of large numbers of transgenic animals, for biological research. Such a methodology could be critical for the recovery of species and their genetic diversity, contributing to amphibian conservation. However, this approach raised the question of whether the sperm preservation method used with ICSI is compatible with long-term storage. To address this question, animals were generated by ICSI using a twenty-year-old cryopreserved sperm preparation. Their development, behavior, and reproduction ability were compared with those of animals obtained using a recently frozen sperm preparation and those of animals obtained via IVF using fresh semen. Although lower than with IVF, we showed that fertilization rates using ICSI after 20 years of cryopreservation are similar to those of a recent preparation, with viable offspring leading to normal F2 generation. This pioneering achievement is proof of concept for long-term sperm cryopreservation using simple and readily available technologies for the conservation of endangered amphibians.

## Simple Summary

Amphibian conservation programs could benefit from the valuable help of assisted reproductive technologies. The objective of the present study was to evaluate the feasibility of using intracytoplasmic sperm injection (ICSI) to generate viable offspring in *Xenopus laevis* with sperm cryopreserved for several years using the freezing method initially developed for transgenesis application purposes in this species. To this end, freshly recovered eggs were fertilized by either ICSI using sperm preparations frozen recently or 20 years ago, or by conventional in vitro fertilization (IVF) using motile fresh sperm. Our results demonstrate that sperm can be cryopreserved for at least 20 years without significant reduction in fertilization capacity using ICSI. Higher proportions of malformations were observed after ICSI using cryopreserved semen when compared to IVF with fresh semen, regardless of the duration of sperm preservation. When metamorphosis was decreased in ICSI-derived tadpoles, particularly when old semen was used, animals showed normal behavior and developed into fertile adults.

## 1. Introduction

Amphibians are the most threatened vertebrate taxon and, as such, a significant component of the sixth mass extinction [1]. Since the 1980s, amphibians have suffered a significant decline in their population worldwide, linked to multifactorial causes such as disease, habitat loss, environmental pollution, the impact of other invasive species, and the effects of climate change. It is estimated that around 40% of existing amphibian species are threatened with extinction according to IUCN data “https://www.iucnredlist.org/resources/summary-statistics (accessed on 30 April 2025)” [1,2,3,4].

This alarming situation has generated a critical demand for the development of breeding programs for the conservation, maintenance, and protection of amphibian species [5,6]. In response, an Amphibian Conservation Action Plan (ACAP) was published for the first time in 2005 to assess this decline and attempt to provide solutions in the form of recommendations to halt the decline of amphibians (“https://www.iucn-amphibians.org/resources/acap/ (accessed on 30 April 2025)”) [7]. As a result, numerous conservation programs have been set up to counteract this decline and preserve amphibian species. However, these programs cannot effectively counter the rate of amphibian decline. A challenge in conservation breeding programs is that some species of amphibians are difficult to breed in captivity.

In 2023, the ACAP document was updated [8], reflecting the evolution of knowledge in the domain of amphibian conservation efforts. The most important change was the addition of a new chapter dedicated to the development of assisted reproductive technologies (ARTs). In recent years, cryobanking and ARTs have become increasingly popular and are important conservation options. Numerous studies are being carried out to develop cryobanking protocols for an increasing number of amphibian species [9]. Currently, ARTs developed for amphibians are essentially based on hormonal induction to harvest oocytes and spermatozoa. For some species, cryopreservation protocols can be performed on sperm for storage and potentially long-term conservation, whereas oocytes cannot be cryopreserved, although they can be stored for hours or days with some species [3,9,10,11]. For some species, the challenge lies in obtaining enough spermatozoa through hormonal stimulation, coupled with cryopreservation of their motility [11,12], and lastly enabling efficient fertilization after thawing. These studies need to (1) define the best protocols to improve sperm collection and the quantity of sperm produced, (2) define the best conditions for cryopreserving the fertilizing capacity of spermatozoa after thawing, and (3) broadly transpose the findings to target species in conservation. This means that for each species tested, the optimal cryopreservation conditions have to be fine-tuned, which is challenging, time-consuming, and costly. For example, in the *Xenopus* species, no less than three methods have been published for sperm cryopreservation, with contrasting results [13,14,15]. Furthermore, applying a similar method in the neighboring species *X. tropicalis* was unsuccessful, and a new protocol was developed [13,15].

In the 1990s, a technology for generating transgenic animals in *Xenopus* was developed [16], enabling reliable production of genetically modified lines in *X. laevis* and *X. tropicalis* species [17,18,19]. This technique is based on a simple and rapid protocol for preparing frozen spermatozoa and storing them in a freezer at −80 °C [16]. The spermatozoa are then thawed and used for intracytoplasmic sperm injection (ICSI) into unfertilized eggs, resulting in egg fertilization and frog development. By adding an exogenous sequence coding for a gene of interest or a reporter to the sperm mixture, this sequence can be inserted into the recipient genome in a significant proportion of offspring, thus creating a genetically modified organism capable of expressing the new gene or the reporter with a particular pattern [16,20,21,22,23,24]. Transgenic animals generated by this method are viable, undergo normal development and metamorphosis, and can reproduce like wild-type (WT) animals. A remarkable advantage of ICSI is that, since fertilization occurs mechanically, the sperm freezing method does not require the preservation of sperm motility. ICSI may thus facilitate the transposition of cryoconservation strategies for a given species.

Optimizing sperm storage and ICSI and their general application is for species conservation by enabling immotile post-thaw sperm to fertilize. These technologies are directly transferable between amphibian species, offering new possibilities for species showing only few motile sperm post thawing. However, ICSI with immotile sperm is a more advanced technology than IVF, requiring greater development of laboratory skills. The optimization of short-term chilling or long-term cryopreservation to maintain spermatozoa integrity is also needed for the setup of the ICSI methodology.

Currently, cryopreserved spermatozoa prepared using this method and used to generate transgenic animals have only been tested over relatively short storage periods at −80 °C. Indeed, ICSI experiments are generally performed with cryopreserved sperm preparations produced as recently as possible in order to increase the chances of generating viable embryos. Thus, long-term data are lacking to determine whether these frozen sperm preparations can be stored for several years, even decades, without affecting sperm quality. In the present study, we therefore propose to test whether this method of sperm preparation and conservation is compatible with prolonged storage periods without losing the ability to produce viable, fertile animals.

## 2. Materials and Methods

### 2.1. Experimental Design

The impact of storage time was assessed on sperm preparations cryopreserved according to the procedure described by Kroll and Amaya in *Xenopus* [16,17,18] and stored at −80 °C. To this end, the ability to generate normally developing individuals either with 20-year-old or less than one-year-old sperm preparations was assessed.

After thawing, intracytoplasmic sperm injection (ICSI) using these sperm preparations was carried out using unfertilized eggs. ICSI experiments with a frozen sperm preparation used after a short storage period are known to result in a high proportion of animals developing without morphological defects. In vitro fertilization (IVF) experiments were performed using fresh semen as control. After fertilization, different parameters such as developmental competence, mortality, the timing of animals’ development, the fate of tadpoles, sex ratio, and animals’ behavior were assessed the experimental conditions. In order to assess whether the animals obtained by ICSI with the old cryoconserved sperm preparation were fertile and capable of producing viable offspring, several couples of adults from the F1 generation were selected and crossed.

### 2.2. Animals and Ethic Statement

The care and treatment of animals were in accordance with institutional and national guidelines (Commission de Génie Génétique, “Direction Départementale des Services Vétérinaires”, European Union Directive 2010/63/EU, agreement decision no. D 75-05-01-2 for the European Convention for vertebrate animals used for experimental and other scientific purposes). All protocols used in this study were approved under the reference number APAFIS #26794 by the Ethical Committee of the National Museum of Natural History (CEEA Cuvier, Paris, France). Wild-type males and females of *Xenopus laevis* used in this study were purchased from the *Xenopus* Biological Resource Center (TEFOR Paris-Saclay, Paris, France).

### 2.3. Animal Growth, Breeding, and Egg Production

Animals from tadpoles to adult frogs are staged according to Nieuwkoop and Faber (NF) nomenclature [25,26]. Animals were housed in a facility with 12:12 h light/dark cycles, 18–20 °C ambient temperature, dechlorinated and filtered water, and a commercial diet adapted depending on age: tadpoles were fed daily with Spiruline (Awwaou, Lormont, France) mixed with SERA micron (Europrix, Lens, France), while the adults were fed three times a week with Tetra Cichlid granules (Europrix, Lens, France) and bloodworms (Europrix, Lens, France). Reproduction was achieved by human chorionic gonadotropin (hCG) stimulation (CHORULON 5000 IU, Centravet, Plancoet, France) of an adult female *X. laevis* followed by nocturnal contact with a mature male. Fresh egg production was achieved using similar hCG stimulation of adult females the evening before egg collection.

### 2.4. Sperm Freezing Preparation and Storage

The old frozen sperm preparation (kindly obtained from Dr. Y.B. Shi laboratory) and the recent frozen sperm preparation were carried out as follows: Frozen sperm from mature male wild-type frogs were prepared using the same procedure described by Kroll and Amaya to perform transgenesis in *Xenopus* [16,17,18]. Briefly, the testes were collected from a euthanized male and macerated with forceps on ice. The suspension was washed and filtered to remove cellular debris, then centrifuged and resuspended in a freezing medium consisting of 1× Nuclear Preparation Buffer (1× NPB: saccharose 250 mM; HEPES 15 mM, pH 7.7; spermidine trihydrochloride 0.5 mM; spermine tetrahydrochloride 0.2 mM; DTT 1 mM; EDTA 1 mM), 0.3% BSA, and 30% glycerol. The sperm suspension was aliquoted, then frozen and stored at −80 °C.

### 2.5. Intracytoplasmic Sperm Microinjection (ICSI)

After thawing and diluting 5 µL of the recent or old frozen sperm preparation in 150 µL of sperm dilution buffer at room temperature (SDB: saccharose 250 mM; KCl 75 mM; spermidine trihydrochloride 0.5 mM; spermine tetrahydrochloride 0.2 mM), spermatozoa were microinjected into dejellied unfertilized eggs using the protocol described for the transgenesis method [16,17,18]. The fertilized eggs were then sorted at the 2–4-cell stage and placed in a 0.1× MMR solution for a week before being maintained in dechlorinated and filtered water. The development of the embryos was monitored until the tadpoles metamorphosed and reached the adult stage.

### 2.6. In Vitro Egg Fertilization (IVF)

Fresh pieces of testis from a euthanized mature male wild-type frog were macerated with forceps and diluted in 0.4× MMR (NaCl 100 mM; KCl 2 mM; MgCl_2_ 1 mM; CaCl_2_ 2 mM; HEPES 5 mM, pH 7.5), allowing for sperm activation, and added for 10 min to eggs for in vitro fertilization (IVF). Fertilized eggs were placed in a 0.1× MMR solution for a week before being maintained in dechlorinated and filtered water; then, the developing embryos were monitored until the tadpoles metamorphosed and reached the adult stage.

### 2.7. Tadpole Behavior/Mobility

At 8 and 22 days post-fertilization (dpf), tadpoles issued from intracytoplasmic sperm microinjection (ICSI) and in vivo egg fertilization (IVF) experiments using eggs from the different females were placed individually into a 12-well plate containing 4 mL of water per well (for 8 dpf tadpoles) or a 6-well plate containing 8 mL of water per well (for 22 dpf tadpoles) and left to acclimate for 15 min. The behavior of the tadpoles was recorded by using a DanioVision video tracking system (11.5, Noldus, Wageningen, The Netherlands) during a 2 min trial composed of two 30 s light-on/30 s light-off intervals, and a brief tapping stimulus at the start of each interval (i.e., 4 tappings were triggered, 2 at the start of light cycles and 2 at the start of dark cycles). During the light-on phase, the maximal light stimulus (5 K Lux) was set. The mobility of each tadpole was calculated by using EthoVision software XT (11.5). For each female tested, the mean distance travelled by the tadpoles in each group was recorded every 2 s.

### 2.8. Statistical Analyses

The collected data from ICSI or IVF experiments were analyzed using Excel (Microsoft) and represented as the mean ± SEM or SD (indicated in tables or graphs captions). Outliers were identified and eliminated with the ROUT test method (Q = 1%), before performing adapted statistical analyses (GraphPad Prism 7.0). The statistical tests used are indicated/described in the figure and table legends when needed.

## 3. Results

### 3.1. ICSI Using an Old Cryoconserved Sperm Preparation Gives Rise to Normal Larval Development Without Phenotypic Defects

To assess the impact of storage time on sperm cryopreservation using the freezing protocol developed by Kroll and Amaya for *Xenopus* transgenesis [16], we tested the ability to generate normally developing animals from sperm preparations stored at −80 °C for twenty years or less than one year.

**Figure 1:**
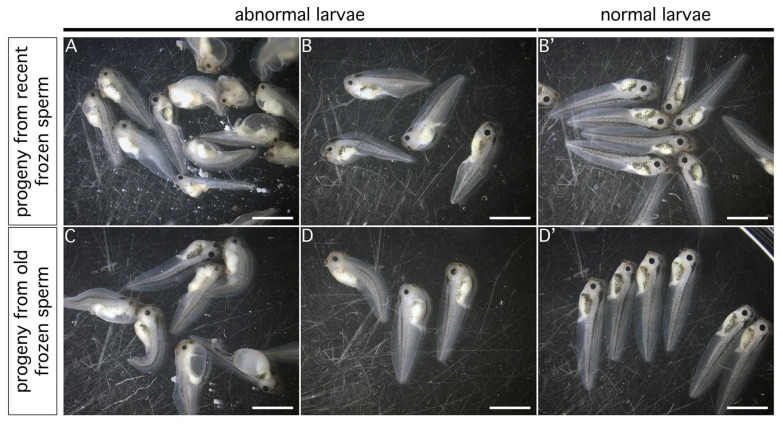
The sorting of larvae according to normal versus abnormal development following ICSI experiments using a recent (**A**,**B**,**B’**) and an old preparation of frozen sperm (**C**,**D**,**D’**). Unfertilized eggs were injected with a recently frozen sperm preparation (recent preparation; top pictures) or cryopreserved for around 20 years (old preparation; bottom pictures). Each picture represents an example of larvae displaying abnormal development at 1–3 days (**A**,**C**) and 4 days (**B**,**D**) post-injection (dpi), compared with larvae developing normally at 4 dpi (**B’**,**D’**). Scale bars: 250 µM.

Although a proportion of animals displaying developmental malformations was observed in both cases (Figure 1A–D), the results showed that the progeny obtained following ICSI experiments with recent and old cryoconserved sperm preparations were capable of giving rise to larvae with a normal phenotype and no developmental defects (Figure 1B’,D’). Furthermore, both preparations displayed comparable percentages of normal larvae at 4 days post-fertilization (dpf), with 38.28% for experiments performed from the recent preparation and 35.35% for experiments performed from the old preparation (Table 1 and Table S1, compare A and B).

### 3.2. ICSI Using Recent and Old Cryoconserved Sperm Preparations Results in Comparable Animal Development but Is Less Efficient than IVF

The normal development rates of animals up to metamorphosis remained quite similar between progeny issued from ICSI with recent and old frozen sperm preparations. Despite some differences, the percentages of animals showing a normal phenotype at the different developmental stages monitored were comparable within the ICSI groups (Table 1 and Table S1, compare A and B). However, the percentages of animals showing a normal phenotype obtained via IVF using fresh semen were significantly higher at all developmental stages monitored (Table 1 and Table S1, compare A and B with C). Indeed, while the rate of fertilized eggs was increased in IVF vs. ICSI groups (with almost 43.5% of dividing eggs vs. 32.4% and 33.7% for ICSI with recent and old preparations, respectively), this shift was significantly reinforced at 4 dpf, with more than 90% vs. 40% of normal larvae observed between IVF and ICSI groups, respectively. Normal development rates were significantly reduced in ICSI groups when compared with IVF for the other stages monitored at 6 dpf (23.65% and 20.25% vs. 65.72% for ICSI vs. IVF groups, respectively, *p* < 0.05) and 15 dpf (20.21% and 17.18% vs. 60.08% for ICSI vs. IVF groups, respectively, *p* < 0.05). However, no effect of sperm preparation was observed on the development calculated from 4 dpf normal animals at 6 and 15 dpf (61.80% vs. 57.27% for recent vs. old preparation at 6 dpf, respectively, *p* = 0.164, and 52.81% vs. 48.60% for recent vs. old preparation at 15 dpf, respectively, *p* = 0.204). Finally, a significant decrease was observed in metamorphosis rate in both ICSI vs. IVF groups (25.59% and 16.57% vs. 36.12% for recent and old preparations vs. IVF groups, respectively, *p* < 0.005). This effect was significant between ICSI semen preparations (*p* < 0.005).

### 3.3. Progeny from Recent and Old Cryopreserved Sperm Preparations Display Comparable Development with Animals Obtained via IVF

**Figure 2:**
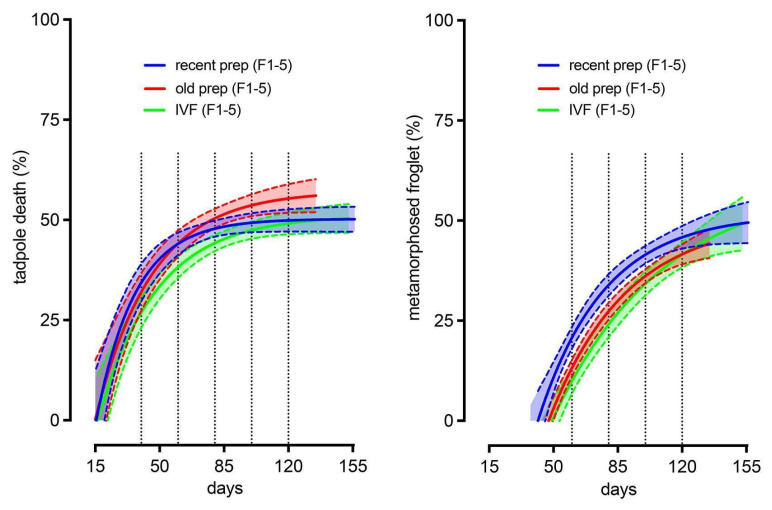
A comparison of tadpole mortality and tadpole development rate curves from ICSI experiments with recent or old cryoconserved sperm preparations and animals obtained through in vitro fertilization (IVF). For each experimental condition, tadpole development is monitored from 15 dpf to the end of metamorphosis and results from 2 independent experiments with the progeny of 5 females per condition (data from individual females are given in Figure S1). Mean% values (full lines) with 95% confidence intervals (light shaded areas inside dashed lines) were used as measures of tadpole death (left graph) or metamorphosed froglets (right graph) for ICSI with recent or old frozen sperm preparations (blue and red curves, respectively) and IVF (green curve). Statistical analysis was performed at 40, 60, 80, 100, and 120 days for tadpole death and 60, 80, 100, and 120 days for metamorphosed froglets (see vertical dashed lines on graphs) by using an ordinary two-way ANOVA followed by Tuckey’s multiple comparisons test. No significant differences (*p* > 0.05) were observed for tadpole death or rate of metamorphosed froglets between the 3 experimental conditions tested.

In each experimental condition, the progeny derived from eggs recovered from five females were analyzed to assess the number of tadpoles that died before the end of metamorphosis; we also analyzed the developmental curve of animals reaching the stage of metamorphosed froglets (Figure 2 and Figure S1).

Graphical representations of animal mortality and development (95% confidence intervals) showed that tadpole death and growth displayed similar profiles with no significant differences observed, regardless of the condition tested (Figure 2). No major differences were observed in the developmental process of IVF-derived animals compared to animals resulting from ICSI experiments, regardless of the sperm preparation employed. Regardless of the conditions, the beginning of metamorphosis occurred among the animals around 50 dpf (Figure 2, and see also Figure S1A–M).

Variability was observed within the experiment. Some delayed metamorphosis was observed for progeny derived from two donor females in the IVF condition (Figure S1N,O). The mortality observed during development was also highly dependent on the female used as the egg donor, regardless of the type of experiment (ICSI or IVF). Indeed, tadpole death displayed a high variability ranging from around 20–30% (Figure S1E,J,M) up to 70–80% (Figure S1B,F,N).

**Figure 3:**
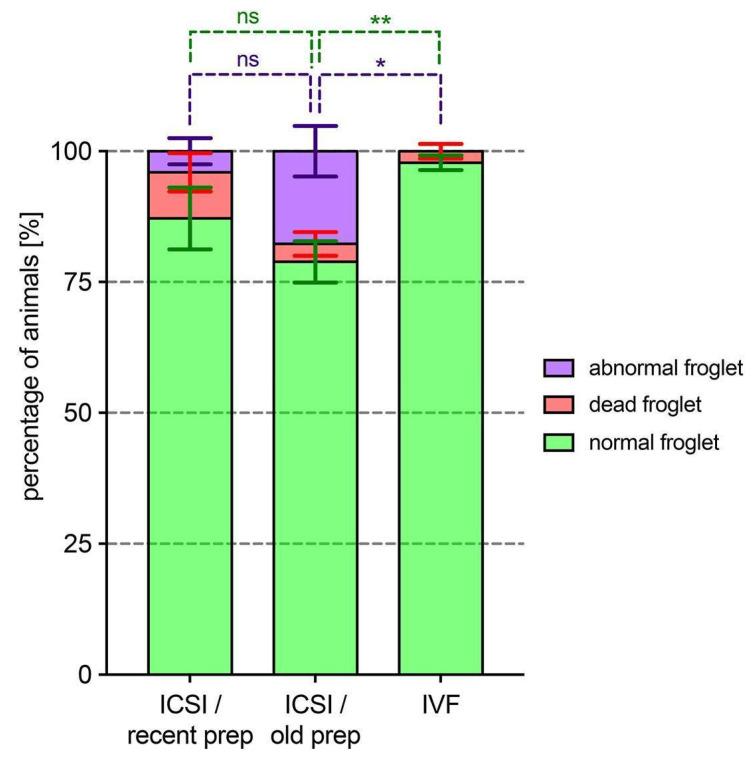
A comparison of froglet fate among ICSI-derived metamorphosed animals with recent or old cryoconserved sperm preparations and metamorphosed animals obtained through in vitro fertilization (IVF). The distribution of normal froglets (in green), abnormal froglets (in purple), and dead froglets (in red) are given for the 3 experimental conditions tested. Data are represented as the mean % value ± SEM for each category and result from 2 independent experiments obtained from the progeny of 5 females per condition (data from individual females are given in Figure S2). Statistical analysis was performed by using an ordinary two-way ANOVA followed by Tuckey’s multiple comparisons test (ns, nonsignificant, *p* > 0.05; *, *p* < 0.05; **, *p* < 0.01). No difference was observed for froglet death between the 3 conditions. Comparing normal (see statistic in green) and abnormal froglets (see statistic in purple), significant differences were observed between IVF and ICSI experiments but not for ICSI performed with recent and old frozen sperm preparations. Note that dead froglets are observed in each condition whereas abnormal froglets are observed in ICSI-derived but not IVF-derived metamorphosed animals.

The mortality rate was compared among metamorphosed froglets, as was the number of animals showing malformations (Figure 3 and Figure S2). We observed that the percentage of normal froglets obtained from ICSI using recent or old frozen sperm preparations did not differ significantly, whereas the percentage of normal froglets obtained via IVF was significantly higher when compared to ICSI experiments (Figure 3). Interestingly, while dead froglets were observed in both ICSI and IVF experiments, froglet malformations were only seen for ICSI-derived animals (Figure 3 and Figure S2). The difference between the percentage of malformed froglets obtained within ICSI preparations was not statistically significant. It should be noted, however, that froglet malformations were systematically observed in the progeny of all the donor females used in the ICSI experiments when the old sperm preparation was used (Figure S2F–J), whereas only the progeny from two of the five females displayed abnormal animal development with the recent sperm preparation (Figure S2A,C).

### 3.4. Sex Ratio Does Not Differ Between Frogs Issued from the Old Cryoconserved Sperm Preparation and Frogs Obtained by Conventional IVF

**Figure 4:**
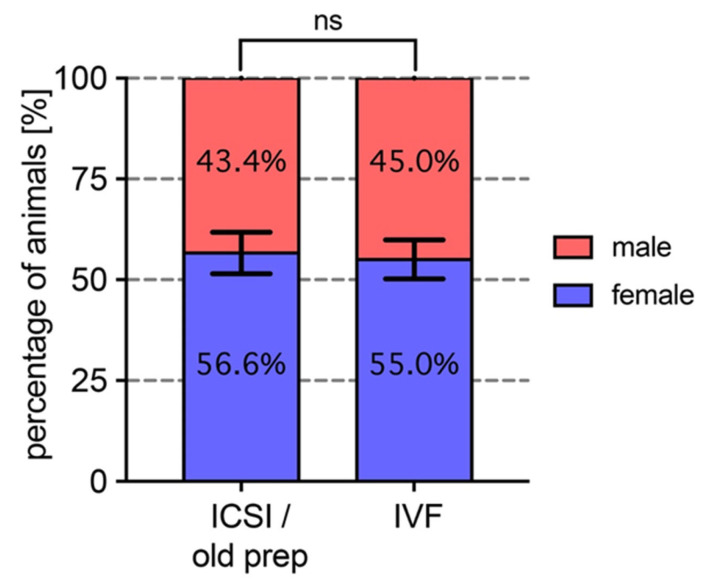
A comparison of the male/female ratio among juvenile animals resulting from ICSI using the old cryoconserved sperm preparation and animals obtained through in vitro fertilization (IVF). Data are represented as the mean % value ± SEM from 2 independent experiments obtained from the progeny of 5 females and 6 females for ICSI and IVF experiments, respectively. Percentages for each male and female categories are indicated in the boxes (data from individual females are given in Figure S3). Statistical analysis was performed by using an ordinary two-way ANOVA followed by Tuckey’s multiple comparisons test. No significant difference (ns, *p* > 0.05) in sex ratio was observed between ICSI and IVF experiments. Note that females are slightly over-represented in both conditions.

The male/female ratio was assessed within the frog population generated by ICSI with the old cryoconserved sperm preparation or obtained via IVF. We assessed the sexual characteristics of 6-month-old juveniles (Figure 4 and Figure S3). This ratio varied according to the origin of the females that provided the eggs used in the ICSI and IVF experiments (see Figure S3 for individual results for each female), but remained very similar between the two experimental conditions, showing a comparable ratio of around 45%/55% for males and females, respectively (Figure 4). In both cases, females were over-represented and in the same proportions in IVF and ICSI experiments.

### 3.5. Behavior Is Comparable Between Tadpoles Issued from Old or Recent Cryoconserved Sperm Preparations and Tadpoles Obtained via IVF

**Figure 5:**
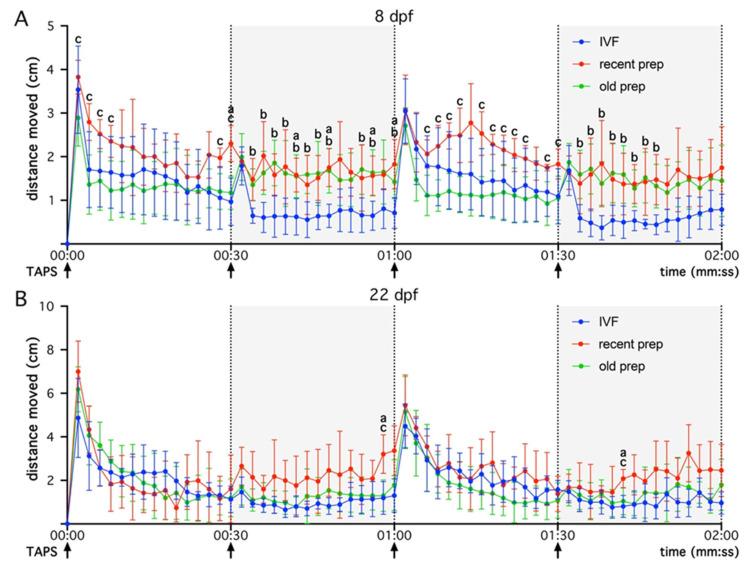
A comparison of tadpole mobility from ICSI experiments with recent or old cryoconserved sperm preparations and animals obtained through in vitro fertilization (IVF). Mobility was measured at 8 days (**A**) and 22 days (**B**) post fertilization (dpf), for 2 min in alternating 30 s light and 30 s dark cycles with brief tapping at the start of each interval (arrows). Distance travelled was analyzed over time. Data are shown as the mean ± SD for 4 to 7 females in each experimental condition, resulting from 2 or 3 independent experiments depending the condition (data from individual females are given in Figure S4). Statistical analysis was performed between the 3 conditions using an ordinary two-way ANOVA followed by Tuckey’s multiple comparisons test. Significant differences (*p* < 0.05) are indicated on the graphs as follows: (a) for “IVF vs. recent prep” (8 dpf: 30 s, 42 s, 48 s, 56 s, and 1:00; 22 dpf: 58 s, and 1:42); (b) for “IVF vs. old prep” (8 dpf: 34 s, 36 s, 38 s, 40 s, 42 s, 44 s, 46 s, 48 s, 54 s, 56 s, 1:00, 1:34, 1:36, 1:38, 1:40, 1:42, 1:44, 1:46, and 1:48); and (c) for “recent prep vs. old prep” (8 dpf: 2 s, 4 s, 6 s, 8 s, 28 s, 30 s, 1:06, 1:08, 1:10, 1:12, 1:16, 1:18, 1:20, 1:22, 1:24, 1:28, and 1:30; 22 dpf: 58 s, and 1:42). Comparable profiles are observed for all conditions. Note the more randomized mobility at 8 dpf for ICSI experiments compared to IVF. However, this randomization was no longer present at 22 dpf, with tadpoles displaying similar behavioral profiles in the 3 groups.

The locomotor activity of tadpoles obtained via ICSI and IVF was monitored at 8 and 22 dpf (Figure 5) using a video tracking system. Tadpoles showed less activity during dark periods, but were more active during light periods (Figure 5). Some differences were observed in the distances covered by tadpoles at 8 dpf (Figure 5A) between the three experimental conditions tested. In particular, in both ICSI groups, animals showed more activity than IVF tadpoles during the first dark phase. This effect was also significant for the old preparation vs. the IVF group in the second dark phase at 8 dpf. The tadpoles’ mean activity from the recent preparation was significantly increased when compared to the old preparation during the second light phase at 8 dpf. In contrast, almost no differences were observed in locomotor activities between the three experimental conditions tested at 22 dpf (Figure 5B). With regard to the tapping stimuli, we observed that tadpoles reacted by showing a burst of activity to each tapping event, with the strongest response observed when the tapping was associated with the light period (Figure 5). The first tapping stimulus was associated with the strongest activity in old vs. recent sperm preparation-derived tadpoles at 8 dpf. No differences were observed when comparing the other tapping responses for the three experimental conditions tested, whether at 8 or 22 dpf (Figure 5).

### 3.6. F2 Generation Obtained from Breeding Animals Generated from the Old Cryopreserved Sperm Preparation Shows Normal Growth and Behavior

**Figure 6:**
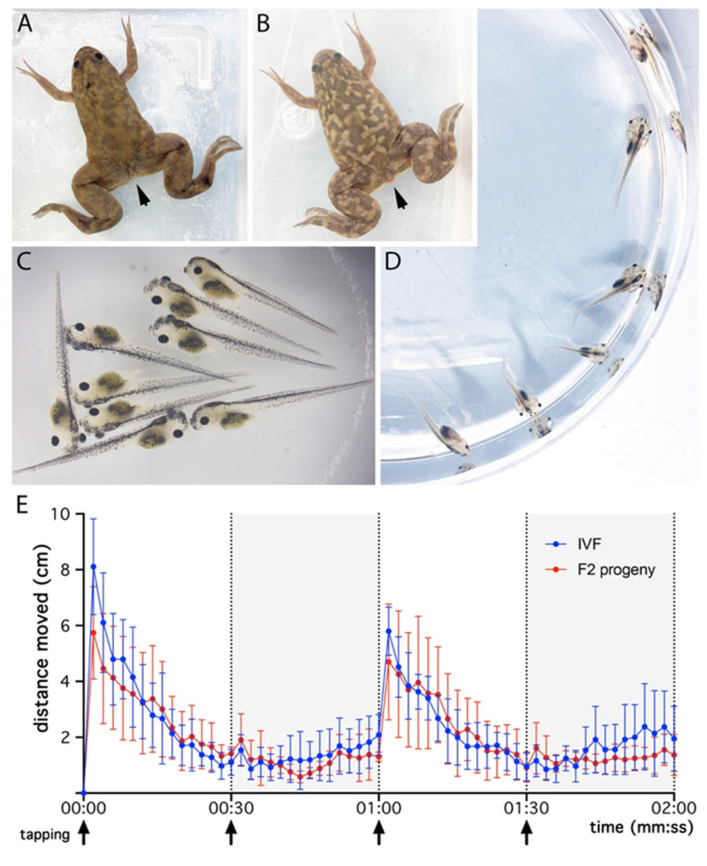
F1 offspring obtained with the old cryoconserved sperm preparation produced viable F2 offspring displaying normal behavior. Crossing an F1 generation adult male (**A**) and female (**B**) derived from ICSI experiments using the old frozen sperm preparation gives rise to an F2 generation showing normal growth and phenotype at larvae (**C**) and tadpole stages (**D**). The arrows in (**A**,**B**) indicate sexual traits that differentiate males from females (with evidence of a more prominent cloaca for the female). Tadpole mobility measured at 22 dpf (**E**) shows no difference between the F2 generation and animals derived from IVF experiments. Mobility was measured for 2 min in alternating 30 s light and 30 s dark cycles with brief tapping at the start of each interval (arrows). Distance travelled was analyzed over time. Data are shown as the mean ± SD for 4 and 6 females in each experimental condition, resulting from 2 or 3 independent experiments (data from individual females are given in Figure S5). Statistical analysis performed between the 2 conditions using an ordinary two-way ANOVA followed by Šídák’s multiple comparisons test showed no significant differences (*p* > 0.05). Note that mobility profiles are highly similar between both conditions.

Several pairs of adult males (Figure 6A) and females (Figure 6B) from the F1 generation were crossed to generate F2 offspring. These crosses resulted in multiple offspring from several couples, giving rise to animals that were viable and showed normal development (Figure 6C,D). In all tested progeny, the tadpoles exhibited normal locomotor activity at 22 dpf (Figure 6E). This locomotor activity was found to be highly similar to that of the progeny obtained via IVF (Figure 6E), and the mobility observed was similar to that in tadpoles from the F1 generation at 22 dpf (Figure 5).

## 4. Discussion

Considering the rapid decline of amphibian species worldwide [4], conservation efforts are urgently needed to reverse the current trend. Habitat protection with management is the most effective conservation strategy for many amphibian species. However, integrating ex situ measures can provide complementary strategies for critically endangered species facing rapid decline from emerging threats such as disease or climate change [27]. For instance, gamete cryobanking and assisted reproductive technologies such as artificial insemination offer significant options for maintaining long-term genetic diversity in such programs [3]. Accordingly, numerous studies are underway to develop efficient sperm cryobanking and insemination protocols for different amphibian models to allow for future restoration of endangered amphibian species [28,29,30,31,32].

Sperm cryopreservation is a crucial step in assisted reproduction techniques for both long-term stabilization of genetics and amphibian conservation [3,33]. The success of in vitro fertilization depends on the ability to maintain the motility and therefore the fertilizing capacity of the spermatozoa after thawing [9,14,34,35]. During sperm cryopreservation and thawing, both cryoprotectant toxicities and cooling/thawing processes are stressful for the spermatozoa, which can impair their motility, survival, DNA integrity, and functionality [36]. As mentioned, several factors need to be considered during cryopreservation, including osmotic stress, the formation of intracellular ice crystals, and the toxicity of cryoprotectants [9,36]. Thus, parameters such as the choice of cryoprotectants, their concentration and duration of exposure, the cooling rate for the freezing phase, and the warming rate during the thawing phase must be systematically validated when developing cryopreservation protocols. The high cost of these approaches hinders the development of conservation programs.

To overcome these difficulties, we assessed the possibility of combining, for amphibian species conservation purposes, a simple sperm conservation protocol with intracytoplasmic sperm injection, which was originally developed to produce transgenic animals in *X. laevis* [16]. By minimizing the need for substituting cryobiology methods, this strategy may represent an interesting complementary approach to conventional sperm freezing programs for ex situ conservation of endangered species. When coupled with intracytoplasmic sperm injection, this alternative method of sperm conservation, focusing on nucleus integrity preservation, does not require the maintenance of the motility of the spermatozoa after thawing. The method has previously been suggested as a conservation tool for transgenic lines [37]. In that study, the authors showed that animals produced via ICSI from such frozen sperm preparations developed normally and exhibited the same characteristics as those of the original line. However, for their demonstration, the authors worked with a short-term cryopreserved sperm preparation before being used for ICSI experiments. The effect of long-term storage of the sperm preparation on fertilization capacity following ICSI was thus not assessed.

The present study was designed to determine whether this simple conservation method could be beneficial for preserving genetics over a long period to re-establish a pool of founder animals. To this end, a sperm preparation stored in a freezer at −80 °C for a period of 20 years was used. Efficiency in producing viable and normal animals via ICSI with an old frozen sperm preparation was compared with an equivalent recently prepared sample. Additionally, we compared our results with those obtained via IVF with fresh semen, which is known to allow for high fertilization rates and a large number of normally developing animals. Our findings demonstrate that the sperm cryoconserved using the freezing method originally developed by Kroll and Amaya can be stored for 20 years without any marked reduction in fertilization capacity. Although different batches of sperm preparations were used to allow for the evaluation of storage time, the results from the present study show 33 and 32% of dividing eggs with old or recent sperm preparations, respectively, suggesting that the storage period did not affect fertilization capacity. The fertilization rates observed are in accordance with previous findings [16,38]. In those studies, the authors reported a global efficiency ranging from 20 to 40% for cleavage rates after ICSI, depending mostly on the quality of the egg recovered. Considering these data, we suggest that it is possible to preserve fertilization capacity over decades using this methodology. In the present study, the progeny obtained via ICSI showed similar development regardless of the frozen sperm preparation employed. The development of these animals was similar to the development observed for animals obtained via control IVF performed with fresh semen. The F1 offspring obtained in all three conditions displayed comparable tadpole development, mortality rates, and lengths of metamorphosis. A comparable sex ratio was also observed among juveniles obtained either from IVF experiments or via ICSI using the old frozen sperm preparation. Considered together, these data suggest the suitability of the method to produce viable offspring.

Nevertheless, some differences were observed between the animals produced via ICSI and those produced via IVF. Indeed, IVF experiments resulted in a higher percentage of animals developing normally and achieving successful metamorphosis than in the ICSI experiments. In addition, metamorphosed animals with malformations were only observed after ICSI but not after IVF fertilization. Furthermore, these malformations were more frequently observed in ICSI-derived animals using the old frozen sperm preparation than the recent one. In amphibians, the developmental competence of the embryo can be defined as the ability to form a larva that develops harmoniously, then induce normal metamorphosis, and finally produce a healthy animal capable of reproducing as an adult. The differences observed within fertilization conditions suggest that IVF is more efficient in preserving embryo developmental competence compared to ICSI, most likely as a result of the mechanical stress associated with intracytoplasmic injection. Although the proportion of malformations remains limited, a significant impact of cryopreservation between sperm preparations or the effect of storage duration on sperm nuclear integrity cannot be excluded. In humans, there is no consensus on whether cryopreservation systematically induces DNA damage, nor on the extent of this damage. While an increase in DNA fragmentation after freezing/thawing has generally been documented in the literature in humans, some data also support the hypothesis that the level of fragmentation could also depend on the quality of the samples collected (donors) or the cryopreservation process (reviewed in [39,40]). However, this phenomenon did not preclude the obtainment of animals with normal development for each ICSI experiment carried out with eggs from different females.

Among ICSI- and IVF-derived animals that displayed normal development, no difference was observed in their behavior. The monitoring of tadpole locomotor activity showed equivalent responses to light and tapping stimuli, with a higher activity during light periods. These results are consistent with observations from previous studies for this species [41,42]. At 22 dpf, the contrast in the amplitudes of the responses between light and dark phases tended to increase for the three experimental conditions. The maturation of the nervous system of tadpoles between 1 and 3 weeks, leading to an improvement in the coordination of the tadpoles’ locomotor movements, could explain this phenomenon. Our data suggest normal development of the tadpoles, including the central nervous system, regardless of the experimental condition assessed.

Finally, a fundamental point to consider for ART development is that the methodology used to cryopreserve sperm not only allows for the production of viable adults but also guarantees that these adults are capable of ensuring the transmission of their genetics to reproduce a new generation of animals [43,44]. By crossing adult F1 males and females obtained from ICSI experiments, we successfully obtained F2 generation animals, which were also able to develop normally. The F2 generation displayed locomotor activity comparable to that of IVF-derived animals. This latest result confirms that sperm cryopreserved for two decades can produce healthy, reproductively competent offspring.

## 5. Conclusions

Our findings demonstrate that cryopreserved sperm can retain remarkable fertilization capacity. Moreover, offspring derived from such sperm preparations using ICSI are viable, develop normally, and can in turn reproduce, highlighting the potential of this approach for amphibian conservation. Interestingly, this method has been also successfully applied to a related species, *X. tropicalis*, giving similar results in terms of efficiency in obtaining viable and fertile offspring [18,19].

In conclusion, our data show evidence that this method may represent an effective alternative for the long-term cryopreservation of WT amphibian spermatozoa. The enrichment of gamete biobanks and the maintenance of the genetic diversity is a major challenge in amphibian conservation, and a simple methodology for sperm preservation would contribute to the development of cryobanking strategies [1]. In the future, extending this technology for application to some criticality endangered amphibian species of anurans, caudata, and gymnophiona would greatly contribute to the conservation of the most imperiled vertebrate taxon, the amphibians.

## Figures and Tables

**Table 1:** A comparison of animal development from ICSI experiments with recent or old cryoconserved sperm preparations and from in vitro fertilization (IVF). The development of the animals was monitored from fertilized eggs to metamorphosed froglets for the 3 experimental conditions tested, and the cumulative frequencies of animals showing correct development are given (data are obtained from at least 3 females per condition, resulting from 2 or 3 independent experiments; details of raw data are given in Table S1). The cumulative frequencies of dividing eggs after ICSI and IVF, relative to the number of eggs injected, are shown in red. Development rates were calculated based on the total number of dividing eggs, or on normal larvae observed at 4 dpf up to metamorphosis. Statistical analyses were performed using *Chi*^2^-tests; values within columns with different superscripts indicate a significant difference at *p* < 0.05.

	Division 0 dpf	Gastrulation at 1 dpf	Normal Animals at 4 dpf	Normal Animals at 6 dpf	Normal Animals at 15 dpf	Metamorphosed Froglet
	From Total	From Cleaved	From Cleaved	From Cleaved		
				(From Normal Animals at 4 dpf)		
In vitro fertilization (IVF)	43.43% ^a^	_	91.53% ^a^	65.72% ^a^	60.08% ^a^	33.06% ^a^
				(71.80%) ^a^	(65.64%) ^a^	(36.12%) ^a^
ICSI with recent frozen sperm preparation	32.41% ^b^	73.55% ^a^	38.28% ^b^	23.65% ^b^	20.21% ^b^	10.58% ^b^
				(61.80%) ^b^	(52.81%) ^b^	(25.59%) ^b^
ICSI with old frozen sperm preparation	33.73% ^b^	75.75% ^a^	35.35% ^b^	20.25% ^c^	17.18% ^b^	5.86% ^c^
				(57.27%) ^b^	(48.60%) ^b^	(16.57%) ^c^

Within a column, values with different superscripts differ significantly at *p* < 0.05.

## Data Availability

The original contributions presented in this study are included in the article. Further inquiries can be directed to the corresponding author(s).

## Supplementary Materials

The following supporting information can be downloaded at https://www.mdpi.com/article/10.3390/ani15131941/s1: Figure S1: Comparison of tadpole mortality and tadpole growth curves from ICSI experiments with recent (A–E) or old cryoconserved sperm preparations (F–I) and animals obtained through in vitro fertilization (IVF; K–O); Figure S2: Comparison of normal vs. abnormal froglets and froglet death among ICSI-derived metamorphosed animals with recent (A–E) or old cryoconserved sperm preparations (F–I) and metamorphosed animals obtained through in vitro fertilization (IVF; K–O); Figure S3: Male/female ratio among juvenile animals resulting from ICSI experiments with old cryoconserved sperm preparation compared to animals obtained through in vitro fertilization (IVF); Figure S4: Comparison of tadpole mobility from ICSI experiments with recent (A,D) or old cryoconserved sperm preparations (B,E) and animals obtained through in vitro fertilization (IVF; C,F); Figure S5: Comparison of tadpole mobility at 22 dpf between F2 generation issued from ICSI experiments using old cryoconserved sperm preparation and animals issued from IVF experiments; Table S1: Monitoring of animal development from ICSI experiments with recent (A) and old cryoconserved sperm preparations (B), and development of animals from in vitro fertilization (IVF; C).

## Abbreviations

The following abbreviations are used in this manuscript:

ICSI	intracytoplasmic sperm injection
IVF	in vitro fertilization
WT	wild type
vs	versus

## References

[1] Browne R.K., Luo Q., Wang P., Mansour N., Kaurova S.A., Gakhova E.N., Shishova N.V., Uteshev V.K., Kramarova L.I., Venu G. (2024). The Sixth Mass Extinction and Amphibian Species Sustainability Through Reproduction and Advanced Biotechnologies, Biobanking of Germplasm and Somatic Cells, and Conservation Breeding Programs (RBCs). Animals.

[2] The IUCN Red List of Threatened Species IUCN Red List of Threatened Species. https://www.iucnredlist.org/en.

[3] Della Togna G., Howell L.G., Clulow J., Langhorne C.J., Marcec-Greaves R., Calatayud N.E. (2020). Evaluating amphibian biobanking and reproduction for captive breeding programs according to the Amphibian Conservation Action Plan objectives. Theriogenology.

[4] Luedtke J.A., Chanson J., Neam K., Hobin L., Maciel A.O., Catenazzi A., Borzée A., Hamidy A., Aowphol A., Jean A. (2023). Ongoing declines for the world’s amphibians in the face of emerging threats. Nature.

[5] Clulow J., Trudeau V.L., Kouba A.J. (2014). Amphibian declines in the twenty-first century: Why we need assisted reproductive technologies. Adv. Exp. Med. Biol..

[6] Clulow J., Upton R., Trudeau V.L., Clulow S., Comizzoli P., Brown J.L., Holt W.V. (2019). Amphibian Assisted Reproductive Technologies: Moving from Technology to Application. Reproductive Sciences in Animal Conservation.

[7] Gascon C. (2007). Amphibian Conservation Action Plan: Proceedings of the IUCN/SSC Amphibian Conservation Summit 2005.

[8] (2024). Amphibian Conservation Action Plan.

[9] Anastas Z.M., Byrne P.G., O’Brien J.K., Hobbs R.J., Upton R., Silla A.J. (2023). The Increasing Role of Short-Term Sperm Storage and Cryopreservation in Conserving Threatened Amphibian Species. Animals.

[10] Browne R.K., Silla A.J., Upton R., Della-Togna G., Marcec-Greaves R., Shishova N.V., Uteshev V.K., Proaño B., Pérez O.D., Mansour N. (2019). Sperm collection and storage for the sustainable management of amphibian biodiversity. Theriogenology.

[11] Guy E.L., Gillis A.B., Kouba A.J., Barber D., Poole V., Marcec-Greaves R.M., Kouba C.K. (2020). Sperm collection and cryopreservation for threatened newt species. Cryobiology.

[12] Germano J.M., Cree A., Molinia F., Arregui L., Bishop P.J. (2022). Hormone treatment does not reliably induce spermiation or mating in Hamilton’s frog from the archaic leiopelmatid lineage. Reprod. Fertil. Dev..

[13] Sargent M.G., Mohun T.J. (2005). Cryopreservation of sperm of *Xenopus laevis* and *Xenopus tropicalis*. Genesis.

[14] Mansour N., Lahnsteiner F., Patzner R.A. (2009). Optimization of the cryopreservation of African clawed frog (*Xenopus laevis*) sperm. Theriogenology.

[15] Pearl E., Morrow S., Noble A., Lerebours A., Horb M., Guille M. (2017). An optimized method for cryogenic storage of *Xenopus* sperm to maximise the effectiveness of research using genetically altered frogs. Theriogenology.

[16] Kroll K.L., Amaya E. (1996). Transgenic *Xenopus* embryos from sperm nuclear transplantations reveal FGF signaling requirements during gastrulation. Development.

[17] Smith S.J., Fairclough L., Latinkic B.V., Sparrow D.B., Mohun T.J. (2006). *Xenopus laevis* transgenesis by sperm nuclear injection. Nat. Protoc..

[18] Ishibashi S., Kroll K.L., Amaya E. (2012). Generating Transgenic Frog Embryos by Restriction Enzyme Mediated Integration (REMI). Methods in Molecular Biology.

[19] Nakayama T., Gray J., Grainger R.M. (2023). Production of Transgenic F0 Animals and Permanent Lines by Sperm Nuclear Transplantation in *Xenopus tropicalis*. Cold Spring Harb. Protoc..

[20] Coen L., du Pasquier D., Le Mevel S., Brown S., Tata J., Mazabraud A., Demeneix B.A. (2001). Xenopus Bcl-XL selectively protects Rohon-Beard neurons from metamorphic degeneration. Proc. Natl. Acad. Sci. USA.

[21] Hirsch N., Zimmerman L.B., Gray J., Chae J., Curran K.L., Fisher M., Ogino H., Grainger R.M. (2002). *Xenopus tropicalis* transgenic lines and their use in the study of embryonic induction. Dev. Dyn..

[22] Coen L., Le Blay K., Rowe I., Demeneix B.A. (2007). Caspase-9 regulates apoptosis/proliferation balance during metamorphic brain remodeling in *Xenopus*. Proc. Natl. Acad. Sci. USA.

[23] Takagi C., Sakamaki K., Morita H., Hara Y., Suzuki M., Kinoshita N., Ueno N. (2013). Transgenic *Xenopus laevis* for live imaging in cell and developmental biology. Dev. Growth Differ..

[24] Paredes R., Ishibashi S., Borrill R., Robert J., Amaya E. (2015). *Xenopus*: An in vivo model for imaging the inflammatory response following injury and bacterial infection. Dev. Biol..

[25] Nieuwkoop P.D., Faber J. (1994). Normal Table of Xenopus laevis (Daudin): A Systematical and Chronological Survey of the Development from the Fertilized Egg Till the End of Metamorphosis.

[26] Zahn N., James-Zorn C., Ponferrada V.G., Adams D.S., Grzymkowski J., Buchholz D.R., Nascone-Yoder N.M., Horb M., Moody S.A., Vize P.D. (2022). Normal Table of *Xenopus* development: A new graphical resource. Development.

[27] Zippel K., Johnson K., Gagliardo R., Gibson R., Browne R., Martinez C., Townsend E. (2011). The Amphibian Ark: A Global Community for Ex Situ Conservation of Amphibians. Herpetol. Conserv. Biol..

[28] Della Togna G., Trudeau V.L., Gratwicke B., Evans M., Augustine L., Chia H., Bronikowski E.J., Murphy J.B., Comizzoli P. (2017). Effects of hormonal stimulation on the concentration and quality of excreted spermatozoa in the critically endangered Panamanian golden frog (*Atelopus zeteki*). Theriogenology.

[29] Poo S., Hinkson K.M. (2020). Amphibian conservation using assisted reproductive technologies: Cryopreserved sperm affects offspring morphology, but not behavior, in a toad. Glob. Ecol. Conserv..

[30] Upton R., Clulow S., Calatayud N.E., Colyvas K., Seeto R.G.Y., Wong L.A.M., Mahony M.J., Clulow J. (2021). Generation of reproductively mature offspring from the endangered green and golden bell frog *Litoria aurea* using cryopreserved spermatozoa. Reprod. Fertil. Dev..

[31] Naranjo R.E., Naydenova E., Proaño-Bolaños C., Vizuete K., Debut A., Arias M.T., Coloma L.A. (2022). Development of assisted reproductive technologies for the conservation of *Atelopus* sp. (spumarius complex). Cryobiology.

[32] Burger I.J., Lampert S.S., Kouba C.K., Morin D.J., Kouba A.J. (2022). Development of an amphibian sperm biobanking protocol for genetic management and population sustainability. Conserv. Physiol..

[33] Zimkus B.M., Hassapakis C.L., Houck M.L. (2018). Integrating current methods for the preservation of amphibian genetic resources and viable tissues to achieve best practices for species conservation. Amphib. Reptile Conserv..

[34] Browne R.K., Kaurova S.A., Uteshev V.K., Shishova N.V., McGinnity D., Figiel C.R., Mansour N., Agnew D., Wu M., Gakhova E.N. (2015). Sperm motility of externally fertilizing fish and amphibians. Theriogenology.

[35] van der Horst G. (2021). Status of Sperm Functionality Assessment in Wildlife Species: From Fish to Primates. Animals.

[36] Hezavehei M., Sharafi M., Kouchesfahani H.M., Henkel R., Agarwal A., Esmaeili V., Shahverdi A. (2018). Sperm cryopreservation: A review on current molecular cryobiology and advanced approaches. Reprod. Biomed. Online.

[37] Buchholz D.R., Fu L., Shi Y.B. (2004). Cryopreservation of *Xenopus* transgenic lines. Mol. Reprod. Dev..

[38] Ishibashi S., Kroll K.L., Amaya E. (2008). A Method for Generating Transgenic Frog Embryos. Methods Mol. Biol..

[39] Di Santo M., Tarozzi N., Nadalini M., Borini A. (2012). Human Sperm Cryopreservation: Update on Techniques, Effect on DNA Integrity, and Implications for ART. Adv. Urol..

[40] Kopeika J., Thornhill A., Khalaf Y. (2015). The effect of cryopreservation on the genome of gametes and embryos: Principles of cryobiology and critical appraisal of the evidence. Hum. Reprod. Update.

[41] Fini J.B., Mughal B.B., Mével S.L., Leemans M., Lettmann M., Spirhanzlova P., Affaticati P., Jenett A., Demeneix B.A. (2017). Human amniotic fluid contaminants alter thyroid hormone signalling and early brain development in *Xenopus embryos*. Sci. Rep..

[42] Leemans M., Spirhanzlova P., Couderq S., Le Mével S., Grimaldi A., Duvernois-Berthet E., Demeneix B., Fini J.B. (2023). A Mixture of Chemicals Found in Human Amniotic Fluid Disrupts Brain Gene Expression and Behavior in *Xenopus laevis*. Int. J. Mol. Sci..

[43] Vance C.K., Julien A., Counsell K., Marcec R., Agcanas L., Tucker A., Kouba A. (2018). Amphibian art over the generations: Frozen sperm offspring produce viable F2 generation. Cryobiology.

[44] Lampert S.S., Burger I.J., Julien A.R., Gillis A.B., Kouba A.J., Barber D., Kouba C.K. (2022). Sperm Cryopreservation as a Tool for Amphibian Conservation: Production of F2 Generation Offspring from Cryo-Produced F1 Progeny. Animals.

