# Supplementary material for: Intracytoplasmic Sperm Injection Using 20-Year-Old Cryopreserved Sperm Results in Normal, Viable, and Reproductive Offspring in Xenopus laevis: A Major Pioneering Achievement for Amphibian Conservation
Source: Animals (Basel). 2025 Jul 1;15(13):1941. doi: 10.3390/ani15131941 (PMC12248916; doi:10.3390/ani15131941)
Supplement: Supplementary file 1 [file animals-15-01941-s001.zip › SUPPLEMENTAL FIGURE & TABLE LEGENDS _with revision.pdf]

SUPPLEMENTAL FIGURE & TABLE LEGENDS

Figure S1: Comparison of tadpole mortality and tadpole growth curves from ICSI experiments with recent (A-E) or old cryoconserved sperm preparations (F-I), and animals obtained through conventional In Vitro Fertilization (IVF; K-O). For each experimental condition, tadpole development is monitored from 15 dpf to the end of metamorphosis; Red and green circles on the graphs indicate every record made over time to follow tadpole death or metamorphosed froglets respectively. Results are shown for 5 females in each experimental condition, and graphs result from 2 independent experiments in each condition. In red is shown the percentage of tadpoles dying during development, and in green the percentage of tadpoles reaching complete metamorphosis (the n indicates the number of animals monitored in each case). Note that the onset of metamorphosis is similar in all 3 cases, the obtention of froglet starting around day 50 post fertilization (50 dpf) independently of the condition tested. Tadpole mortality varies according to female or condition. It remains nonetheless comparable between experiments carried out with the old and the recent frozen sperm preparations, and is slightly lower for animals from IVF performed with fresh sperm.

Figure S2: Comparison of normal vs abnormal froglets and froglet death among ICSI-derived metamorphosed animals with recent (A-E) or old cryoconserved sperm preparations (F-I), and metamorphosed animals obtained through conventional In Vitro Fertilization (IVF; K-O). Data are shown for 5 females in each condition and animals analyzed in each graph correspond to the metamorphosed froglets obtained in Figure S2. For each graph, n represent the number of animals analyzed; For each experimental condition, data result from 2 independent experiments. Green box represents the % of frogs with a normal phenotype, red box the rate of dead frogs after complete metamorphosis and purple box the rate of frogs with abnormal development.

Figure S3: Male/female ratio among juvenile animals resulting from ICSI experiments with an old cryoconserved sperm preparation compared to animals obtained through conventional In Vitro Fertilization (IVF). Data are shown for the progeny of 5 females for experiments done using the old frozen sperm preparation and 6 females for IVF experiments. For each graph, n represent the number of animals analyzed; For each experimental condition, data result from 2 independent experiments.

Figure S4: Comparison of tadpole mobility from ICSI experiments with recent (A and D) or old cryoconserved sperm preparations (B and E), and animals obtained through *in vitro fertilization* (IVF; C and F). Mobility was measured at 8 and 22 days post fertilization (dpf), for 2 min in alternating 30 s light and 30 s dark cycles with a brief tapping at the start of each interval (arrows). Distance travelled was analyzed over time. Data are shown as the mean ± SD for 4 to 7 females in each experimental condition, resulting from 2 or 3 independent experiments depending the condition (For each curve, n= 12 to 36 tadpoles for 8 dpf experiments and n= 6 to 12 tadpoles for 22 dpf experiments).

Figure S5: Comparison of tadpole mobility at 22 dpf between F2 generation derived from ICSI experiments using the old cryoconserved sperm preparation and animals derived from IVF experiments. Mobility was measured for 2 min in alternating 30 s light and 30 s dark cycles with a brief tapping at the start of each interval (arrows). Distance travelled was analyzed over time. Data are shown as the mean ± SD for 4 and 6 females in each experimental condition, resulting from 2 or 3 independent experiments (for ICSI condition, n= 6, 15, 24 and 21 tadpoles per curve for F1 to 4 respectively; for IVF condition, n= 23, 4, 21, 13, 24 and 21 tadpoles per curve for F1 to 6 respectively).

Table S1: Monitoring of animal development from ICSI experiments with recent (A) and old cryoconserved sperm preparations (B), and the development of animals from *in vitro fertilization* (IVF; C). Results are given for 4 to 8 females in each condition, obtained from 2 or 3 independent experiments depending the condition. For each female, upper line indicates the number of injected eggs (for ICSI experiments) or fertilized eggs (for IVF experiments), then the number of dividing eggs, followed by the number of animals developing normally at 1 dpf (gastrulation), 4 dpf, 6 dpf, 15 dpf and up to metamorphosis. The bottom line indicates percentages of eggs dividing after ICSI or IVF, relative to the number of eggs injected or fertilized respectively (% shown in red). For the next 5 columns, calculated on the number of eggs dividing, the percentages of normal embryos at 1 dpf (gastrulation), then 4, 6, 15 dpf and up to metamorphosis are shown (nd: not determined). Data in grey lacking metamorphosis rates (not determined or strong effect of contamination) were not considered for analysis based on cumulative frequencies comparison showed in Table 1. Note that a high abnormal tadpole mortality due to bacterial contamination of the medium has been observed in experiment (see values with ** asterisks for female 4 in C).
