# Supplementary material for: Intracytoplasmic Sperm Injection Using 20-Year-Old Cryopreserved Sperm Results in Normal, Viable, and Reproductive Offspring in Xenopus laevis: A Major Pioneering Achievement for Amphibian Conservation
Source: Animals (Basel). 2025 Jul 1;15(13):1941. doi: 10.3390/ani15131941 (PMC12248916; doi:10.3390/ani15131941)
Supplement: Supplementary file 1 [file animals-15-01941-s001.zip › TABLE S1_revised.pdf]

Table S1

A

ICSI with recent frozen sperm preparation	Number of injected eggs (t=0)	Number of dividing eggs (0dpf)	Number of gastrulating eggs (1dpf)	Number of normal embryos at 4dpf	Number of normal embryos at 6dpf	Number of normal embryos at 15dpf	Number of metamorphosed froglet
♀1	530	195<br>*36,79%*	119<br>*61,03%*	79<br>*40,51%*	57<br>*29,23%*	46<br>*23,59%*	22<br>*11,28%*
♀2	589	198<br>*33,62%*	123<br>*62,12%*	70<br>*35,35%*	58<br>*29,29%*	55<br>*27,78%*	15<br>*7,58%*
♀3	376	126<br>*33,51%*	92<br>*73,02%*	47<br>*37,30%*	29<br>*23,02%*	22<br>*17,46%*	9<br>*7,14%*
♀4	398	86<br>*21,61%*	65<br>*75,58%*	40<br>*46,51%*	16<br>*18,60%*	13<br>*15,12%*	7<br>*8,14%*
♀5	294	117<br>*39,80%*	95<br>*81,20%*	20<br>*17,09%*	14<br>*11,97%*	10<br>*8,55%*	nd<br>*nd*
♀6	682	208<br>*30,50%*	190<br>*91,35%*	100<br>*48,08%*	46<br>*22,12%*	42<br>*20,19%*	33<br>*15,87%*
	Mean:	*32,64%*	*74,05%*	*37,48%*	*22,37%*	*18,78%*	*10,00%*

B

ICSI with old frozen sperm preparation	Number of injected eggs (t=0)	Number of dividing eggs (0dpf)	Number of gastrulating eggs (1dpf)	Number of normal embryos at 4dpf	Number of normal embryos at 6dpf	Number of normal embryos at 15dpf	Number of metamorphosed froglet
♀1	1057	278<br>*26,30%*	160<br>*57,55%*	83<br>*29,86%*	65<br>*23,38%*	57<br>*20,50%*	12<br>*4,32%*
♀2	654	233<br>*35,63%*	174<br>*74,68%*	116<br>*49,79%*	96<br>*41,20%*	90<br>*38,63%*	17 (*)<br>*7,30%*
♀3	586	217<br>*37,03%*	126<br>*58,06%*	50<br>*23,04%*	41<br>*18,89%*	34<br>*15,67%*	17<br>*7,83%*
♀4	963	270<br>*28,04%*	169<br>*62,59%*	69<br>*25,56%*	49<br>*18,15%*	38<br>*14,07%*	16<br>*5,93%*
♀5	1986	838<br>*42,20%*	808<br>*96,42%*	488<br>*58,23%*	466<br>*55,61%*	339<br>*40,45%*	nd<br>*nd*
♀6	2156	829<br>*38,45%*	755<br>*91,07%*	328<br>*39,57%*	119<br>*14,35%*	95<br>*11,46%*	62<br>*7,48%*
♀7	829	294<br>*35,46%*	258<br>*87,76%*	156<br>*53,06%*	135<br>*45,92%*	93<br>*31,63%*	nd<br>*nd*
♀8	163	57<br>*34,97%*	40<br>*70,18%*	21<br>*36,84%*	21<br>*36,84%*	17<br>*29,82%*	nd<br>*nd*
	Mean:	*34,76%*	*74,79%*	*39,49%*	*31,79%*	*25,28%*	*6,57%*

C

In Vitro Fertilization (IVF)	Number of injected eggs (t=0)	Number of dividing eggs (0dpf)	Number of gastrulating eggs (1dpf)	Number of normal embryos at 4dpf	Number of normal embryos at 6dpf	Number of normal embryos at 15dpf	Number of metamorphosed froglet
♀1	285	66<br>*23,16%*	nd	59<br>*89,39%*	36<br>*54,55%*	35<br>*53,03%*	22<br>*33,33%*
♀2	148	118<br>*79,73%*	nd	109<br>*92,37%*	91<br>*77,12%*	84<br>*71,19%*	38<br>*32,20%*
♀3	138	64<br>*46,38%*	nd	59<br>*92,19%*	36<br>*56,25%*	30<br>*46,88%*	22<br>*34,38%*
♀4	222	68<br>*30,63%*	nd	65<br>*95,59%*	56<br>*82,35%*	27 (**)<br>*39,71%*	5 (**)<br>*7,35%*
	Mean:	*44,97%*	*nd*	*92,39%*	*67,57%*	*52,70%*	*26,82%*

(*) bacterial contamination; (**) important death due to bacterial contamination; nd: not determined.
