# Supplementary figures and images for: Intracytoplasmic Sperm Injection Using 20-Year-Old Cryopreserved Sperm Results in Normal, Viable, and Reproductive Offspring in Xenopus laevis: A Major Pioneering Achievement for Amphibian Conservation
Source: Animals (Basel). 2025 Jul 1;15(13):1941. doi: 10.3390/ani15131941 (PMC12248916; doi:10.3390/ani15131941)

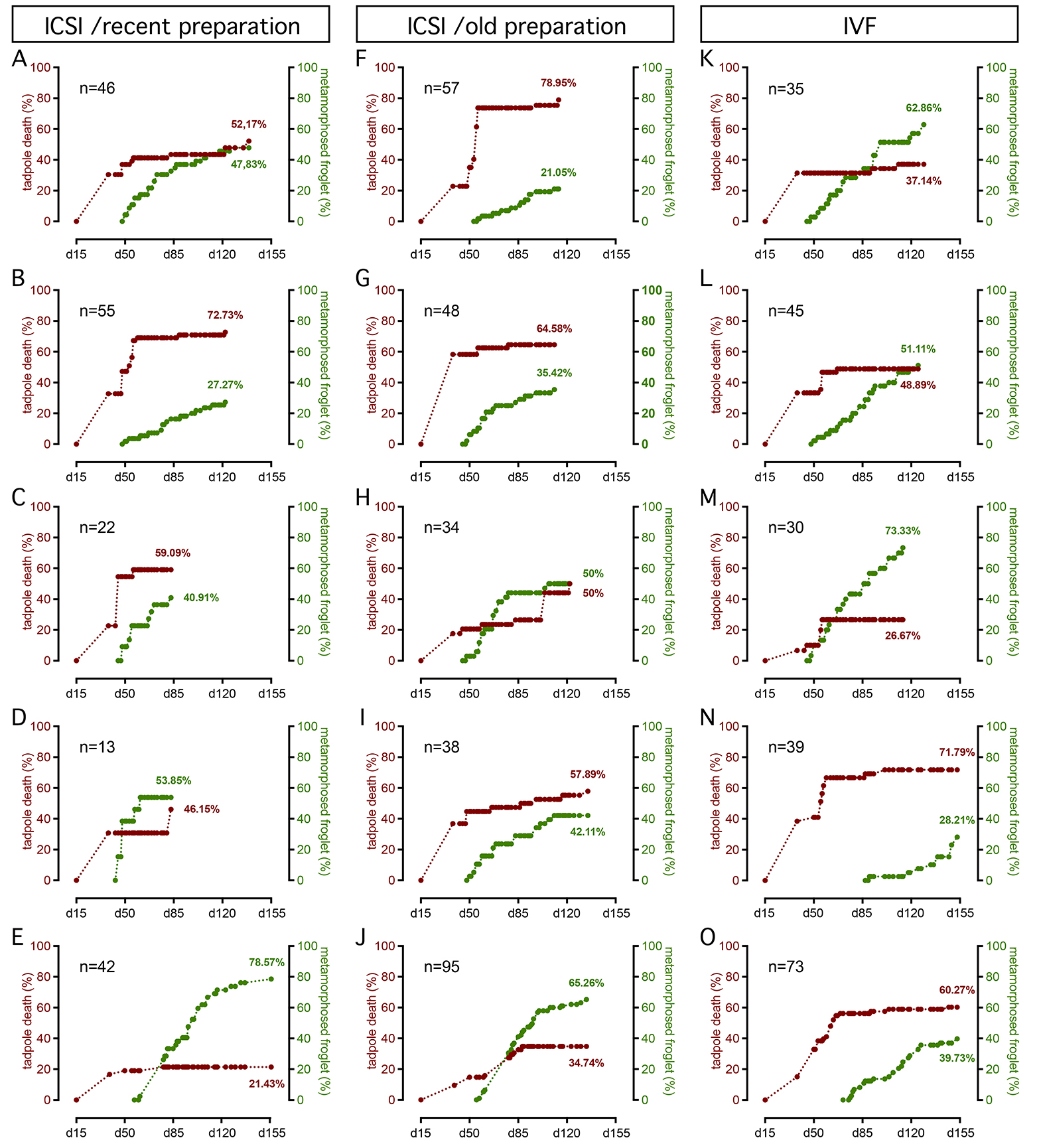

Supplement: Supplementary file 1 [file animals-15-01941-s001.zip › FIGURE S1.jpg]

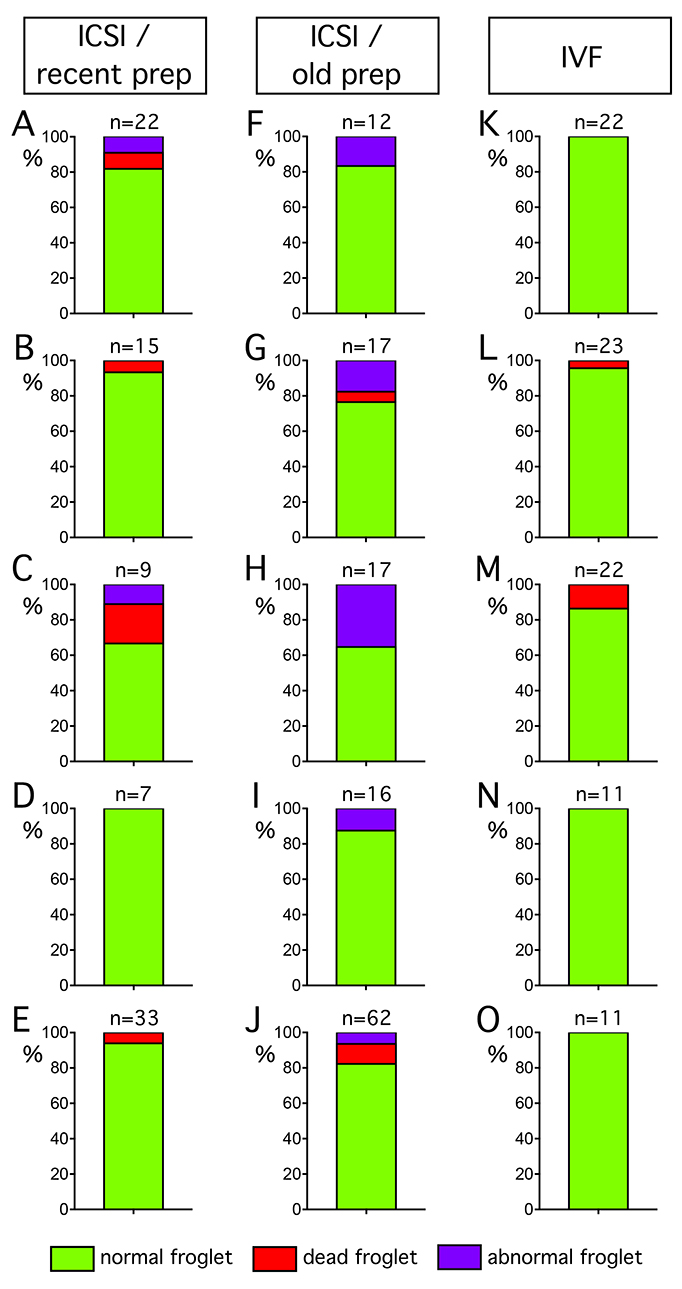

Supplement: Supplementary file 1 [file animals-15-01941-s001.zip › FIGURE S2.jpg]

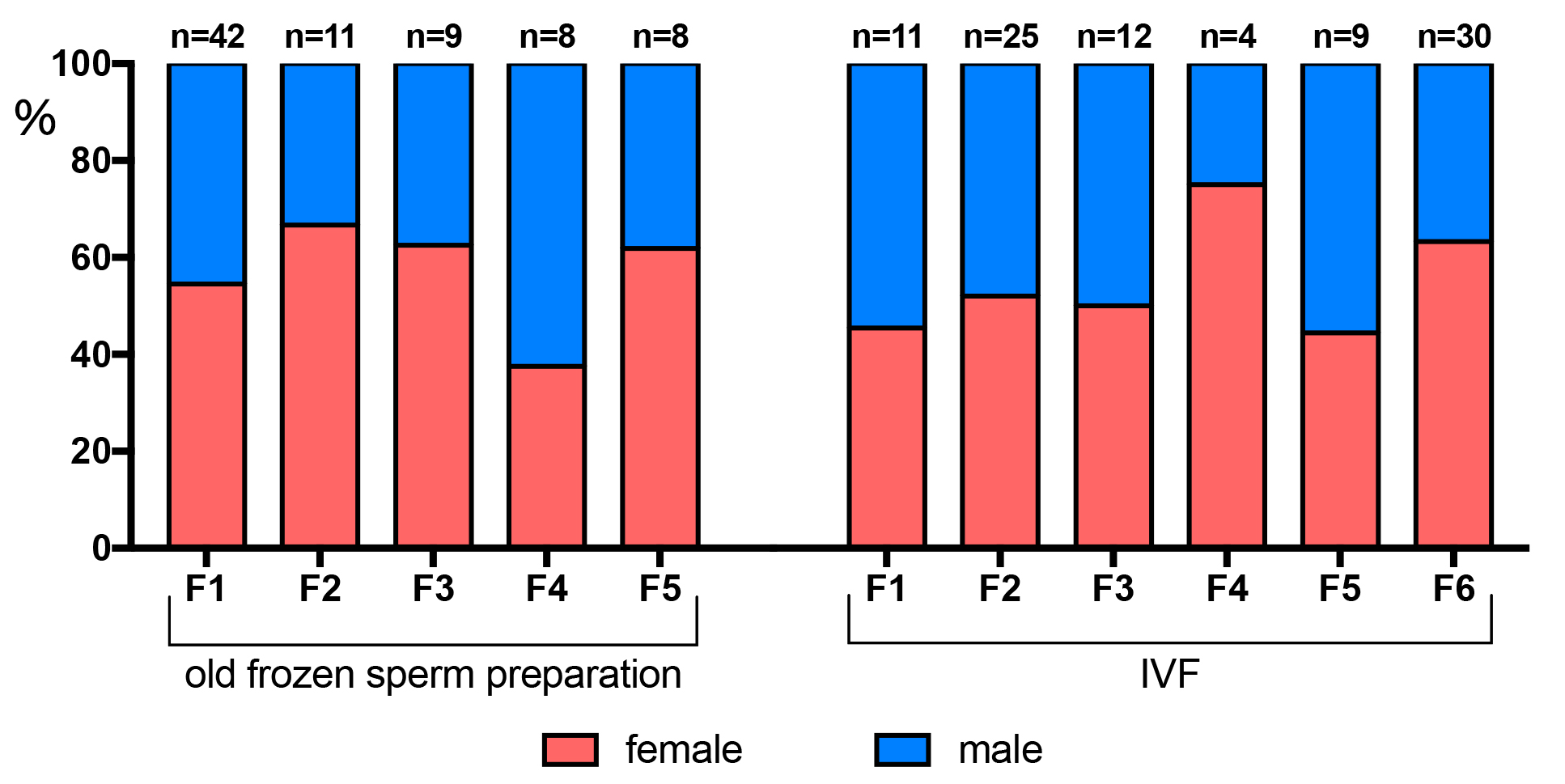

Supplement: Supplementary file 1 [file animals-15-01941-s001.zip › FIGURE S3.jpg]

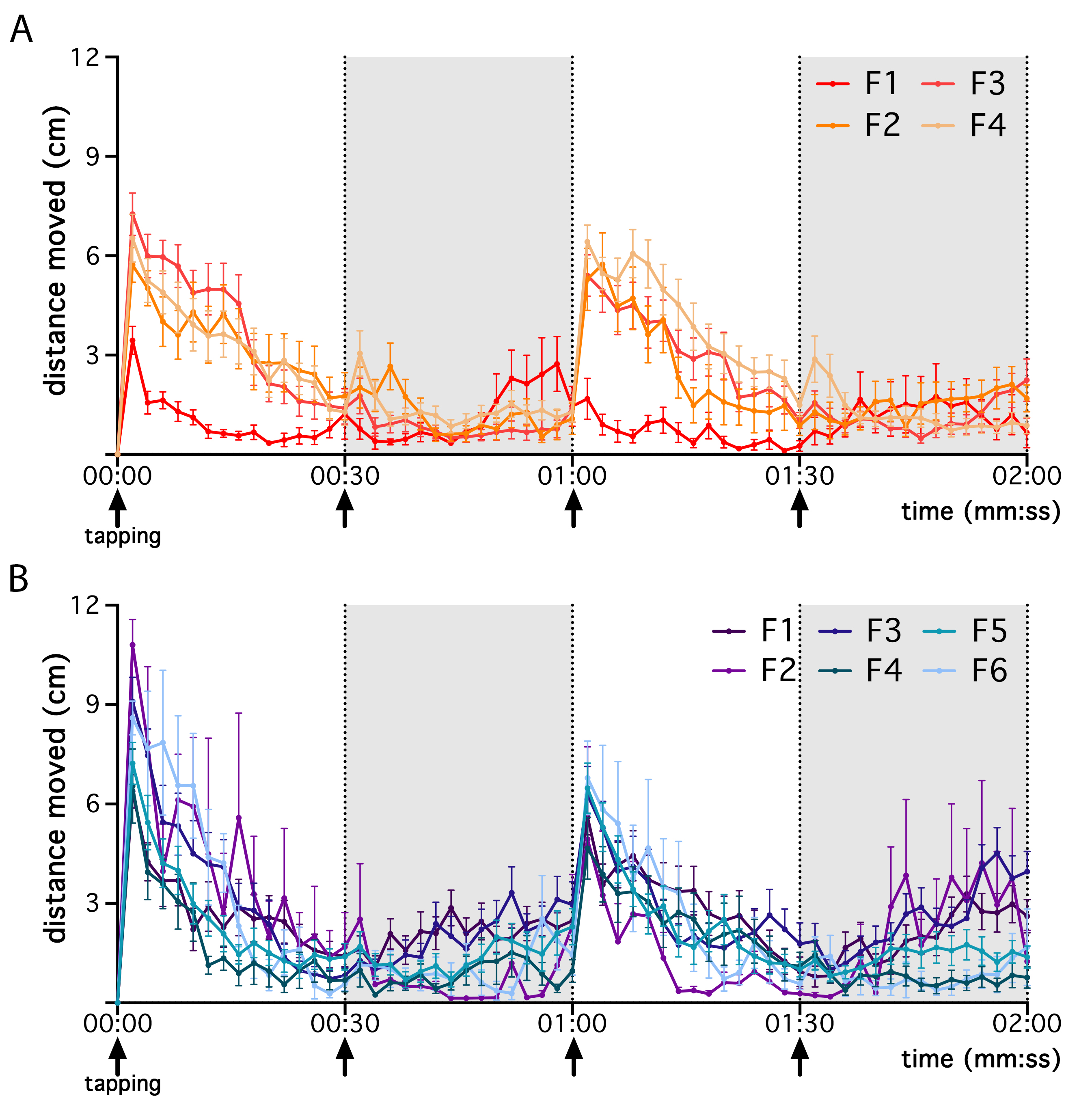

Supplement: Supplementary file 1 [file animals-15-01941-s001.zip › FIGURE S5_revised.jpg]
